# Knowledge of and Barriers to Palliative Care Perceived by Healthcare Providers before and after Promotion of the Patient Autonomy Act: A Cross-Sectional Study

**DOI:** 10.3390/ijerph19073884

**Published:** 2022-03-24

**Authors:** I-Hui Chen, Shu-Fen Kuo, Yen-Kuang Lin, Tsai-Wei Huang

**Affiliations:** 1 School of Nursing, College of Nursing, Taipei Medical University, Taipei 11031, Taiwan; ichen4@tmu.edu.tw (I.-H.C.); sfkuo6@tmu.edu.tw (S.-F.K.); 2 Graduate Institute of Athletics and Coaching Science, National Taiwan Sport University, Taoyuan 33301, Taiwan; robbinlin@ntsu.edu.tw; 3 Cochrane Taiwan, Taipei Medical University, Taipei 11031, Taiwan; 4 Center for Nursing and Healthcare Research in Clinical Practice Application, Wan Fang Hospital, Taipei Medical University, Taipei 11696, Taiwan; 5 Department of Nursing, Wan Fang Hospital, Taipei Medical University, Taipei 11696, Taiwan

**Keywords:** barrier, healthcare provider, knowledge, palliative care, Patient Autonomy Act

## Abstract

This study was designed to investigate healthcare providers’ knowledge of palliative care and perceptions of palliative care barriers before and after promoting the Patient Autonomy Act (PAA). A convenience sample was recruited, including 277 healthcare providers in 2013 and 222 healthcare providers in 2018. Multivariate linear regression analyses were used to identify predictors of knowledge of and perceived barriers to palliative care. A principal component analysis was carried out to identify the most appropriate factorial structure for the contents of knowledge and perceived barriers to palliative care. Three factors related to knowledge of palliative care were identified in both 2013 and 2018 data: ‘policy, regulation, and promotion’, ‘philosophy and treatments’, and ‘myths and misunderstandings’. Study findings for the two periods were similar. As for barriers to providing palliative care, three factors were identified for 2013: ‘quality care’, ‘difficulties’ and ‘communication’, and for 2018, ‘information’, ‘attitudes’ and ‘quality care’ were identified. Study findings differed between the two periods. Policies can better reinforce mitigating strategies—including opportunities for education, shared decision making, and changes in institutions and care systems. Additionally, assessing barriers creates important opportunities for further research to address the most critical aspects in improving end-of-life care for patients and their families.

## 1. Introduction

Demographic projections indicate that by 2026, one in five Taiwanese will be at least 65 years of age [1], suggesting that an increasing number of people will be living with terminal conditions and could benefit from palliative care since one of the ultimate goals of palliative care is to achieve a good death [2]. Many Western countries have laws about decision making at the end of life, such as the Patient Self-Determination Act in the US [3] and the Mental Capacity Act in the UK [4]. Taiwan is among the group of countries where hospice palliative care services are at a stage of advanced integration into mainstream service provision based on criteria set by Lynch, Connor, and Clark [5].

In 2000, Taiwan became the first country in Asia to enact legislation, the Hospice Palliative Care Act, related to the controversial topic of natural death. It was enacted for the purpose of respecting medical wishes of cancer patients at the terminal stage and safeguarding their rights [2,6,7,8]. In 2016, the Patient Autonomy Act (PAA) was passed, and it was enacted in January 2019. The PAA is similar to the Patient Self-Determination Act. The PAA expands upon the existing Hospice Palliative Care Act and covers a wider range of conditions. The PAA allows all patients to establish an advance decision to decide what kinds of life-sustaining treatment and artificial nutrition and hydration they would refuse in the future when they are experiencing one of the five following clinical conditions: a terminal illness, an irreversible coma, a permanent vegetative state, severe dementia, and other disease conditions announced by the central competent authority [9]. Additionally, what sets the PAA apart from the Hospice Palliative Care Act is that it ensures that a patient has the right to be informed regarding their treatment options and decisions regarding that treatment. Based on the PAA, palliative consultations are required to be given to patients before they sign the documents regarding life-sustaining treatments. Through advanced care planning (ACP), patients may sign advance directions (ADs). The ADs shall be made by a person ≥20 years old and has legal capacity without being subject to an order of commencement of guardianship [9]. The PAA recommends that if physicians or medical organizations do not comply with a patient’s advanced wishes, the patient can be transferred to another hospital. Taiwan is the first country in Asia to pass legislation of this kind to protect and respect patient rights to a good death [10].

The PAA must have impacts on healthcare providers (HPs). Physicians normally do not explain to cancer patients or those whose lives are in danger what condition they are in; instead, they explain and discuss this with the patient’s family members [11,12]. Additionally, HPs’ conventional obligation was previously to save a person’s life instead of assisting with their death. Having respect for a patient’s autonomous right to choose a good death, the PAA mandates that HPs assist a patient’s death according to his/her own will. Accordingly, HPs are supposed to be completely adjusted to this condition.

HPs’ perceptions about palliative care will influence their execution of the PAA. HPs play major roles in influencing patients’ and families’ awareness of palliative care [2]. Barriers to providing optimal palliative care have been identified in the existing literature, including an uncertain prognosis, family preferences for more life-sustaining treatment, insufficient knowledge of palliative care [13,14], and a lack of training and expertise [13,15]. In view of this, during the period of 2016~2018, many efforts were made to promote the PAA, such as nationally producing many advertisements and conducting campaigns and in-service training programs for HPs [16]. However, no study has evaluated the impacts of this effort to promote the PAA on HPs. This study was designed to measure HPs’ knowledge of palliative care and perceptions of palliative care barriers from the viewpoints of HPs caring for patients. This study investigated two research questions, namely: (1) What demographic characteristics influenced HPs’ knowledge of palliative care and perceptions of barriers to providing palliative care before and after promoting the PAA? and: (2) How did HPs’ knowledge of palliative care and barriers that limited their provision of palliative care change from before promoting the PAA to after promoting the PAA?

## 2. Materials and Methods

### 2.1. Design

This study had a cross-sectional design using a self-reported anonymous questionnaire to examine HPs’ (physicians’ and nurses’) knowledge of palliative care and barriers they encountered while providing palliative care. Surveys were conducted in 2013 (2013 survey) and 2018 (2018 survey). We used the 2018 survey year as a proxy to examine the effects of promoting the PAA. 

### 2.2. Participants

Participants were recruited from two regional hospitals located in central and northern Taiwan. For a medium effect size (d = 0.3), a power of 0.8, and a significance level of 0.05, a minimum of 220 participants is required. Inclusion criteria were: (1) aged ≥20 years; (2) licensed physician, registered nurse, or licensed practical nurse; (3) working in current hospital ≥6 months; (4) able to read Chinese; and (5) willing to participate in this study. To comprehend the knowledge of and barriers to palliative care perceived by general HPs, the study was not restricted to those with experience in caring for dying patients. Exclusion criteria were students in their internship period and HPs who took their leave. In total, a convenience sample of 277 HPs (62 physicians and 215 nurses) from the hospital in central Taiwan in 2013 were approached, and all agreed to participate in the study. In 2018, a total of 224 HPs were approached, but two participants did not respond; hence, only 222 HPs (53 physicians and 169 nurses) from the hospital in northern Taiwan in 2018 were recruited.

### 2.3. Instruments

Survey questions were organized into three sections: knowledge of palliative care, perceived barriers to palliative care, and demographics. The scales of knowledge of palliative care and perceived barriers to palliative care were developed based on an extensive review of the literature and the authors’ own clinical experience. Knowledge of palliative care scale is composed of 30 items, promotion, myths, and misunderstandings, relevance to medical philosophy and treatments in palliative care, informed consent and ADs, and policies and regulations. Four items from the myths and misunderstandings category were reverse items. The perceived barriers to palliative care scale is composed of 32 items, patient and family attitudes, messaging and communication, HPs’ attitudes, HPs’ ability and training, patient and family knowledge, HPs’ knowledge, and relevance to policy resources and economics. Both scales use a 5-point Likert scale (1 = totally disagree; 5 = totally agree). A higher score indicates a greater knowledge of palliative care or the presence of more barriers to palliative care. The content validity analysis of the scales of knowledge of palliative care and perceived barriers to palliative care showed a global content validity index (CVI) of 0.96 and 0.97, respectively. Additionally, the Cronbach’s alpha of the scales of knowledge of palliative care and perceived barriers to palliative care revealed coefficients of 0.92 and 0.95, respectively. The two scales had good validity and reliability [17]. Demographics included age, gender, job tenure, marital status, educational level, and working unit.

### 2.4. Ethical Considerations

The institutional review board approved the project proposal prior to the initiation of the 2013 study (HP 110002) and another study in 2018 (1-106-05-163). HPs were informed about the purpose of the study and the voluntary participation and confidentiality of data, and returning the completed questionnaire represented their consent. This study complied with all ethical guidelines for human experimentation stated in the Helsinki Declaration.

### 2.5. Statistical Analyses

Demographics were reported as the mean, standard deviation (SD), and proportion. Multivariate linear regression analyzes were used to identify predictors of knowledge of and perceived barriers to palliative care. All demographic factors and perspectives were included in the univariate analyses. Those for which the results were significant were then included in the multivariable analysis. A principal component analysis was employed, allowing us to identify the most appropriate factorial structure for contents of knowledge of palliative care in 2013 and 2018, as well as for contents of perceived barriers to palliative care in 2013 and 2018. Moreover, two-sample t-test was performed to compare the total scores and subscores of the knowledge and barriers scales in 2013 and 2018. *p*-values were two-sided and considered significant at <0.05. Statistical analyzes were performed with IBM SPSS 22.0 (IBM Corporation. IBM SPSS, Armonk, NY, USA).

## 3. Results

### 3.1. Characteristics of Participants

As shown in Table 1, in the 2013 survey, the average age of participants was 33.2 (SD = 8.1) years. The majority were female (79.4%), had working experience of over a decade (36.1%), were unmarried (50.2%), had a bachelor’s degree (62.8%), and worked in the medical or surgical ward (58.5%). Mean scores for knowledge of palliative care and perceived barriers to palliative care were 112 (SD = 15.0, range = 85~150) and 106.4 (SD = 18.2, range = 66~160), respectively. In the 2018 survey, the average age of participants was 32.1 (SD = 8.5) years. The majority were female (78.8%), had working experience of over a decade (33.3%), were unmarried (50.0%), had a bachelor’s degree (61.7%), and worked in the medical or surgical ward (49.5%). Mean scores for knowledge of palliative care and perceived barriers to palliative care were 115 (SD = 14.6, range = 79~145) and 99.6 (SD = 15.8, range = 66~157), respectively. 

### 3.2. Predictors of Knowledge of Palliative Care and Perceived Barriers to Palliative Care

Multivariable linear regression analyses revealed factors associated with knowledge and barriers in 2013 and 2018. In Table 2, no variables were significantly related to knowledge of palliative care in 2013. Having a junior college degree (B = −0.309, *p* = 0.03) and a bachelor’s degree (B = −0.298, *p* = 0.03) were significantly related to knowledge of palliative care in 2018. 

Age (B = −0.019, *p* = 0.03) and job tenure (B = 0.119, *p* = 0.001) were significantly related to perceived barriers to palliative care in 2013. Having a junior college degree (B = −0.562, *p* = 0.0008) and a bachelor’s degree (B = −0.376, *p* = 0.02) predicted perceived barriers to palliative care in 2018 (see Table 3).

### 3.3. Changes in Knowledge of Palliative Care and Perceived Barriers to Palliative Care

A three-factor model gave an adequate description of the data of knowledge of palliative care. In 2013 data (see Table 4), most items with salient loadings on factor 1 contained a variety of non-overlapping policies and regulations and promotion. This factor was labeled ‘policies, regulations, and promotion’. Factor 2 contained a variety of items describing different aspects of medical philosophy and palliative care treatments. This factor was labeled ‘philosophy and treatments’. Factor 3 contained items describing myths and misunderstandings, informed consent, and ADs. This factor was labeled ‘myths and misunderstandings’. In 2018 data, the pattern of factor 1 was the same as that of factor 1 in 2013. This factor was labeled ‘policies, regulations, and promotion’. Factor 2 was still labeled ‘philosophy and treatments’. This factor contained not only items of medical philosophy and treatments in palliative care, but also items describing informed consent and ADs. Factor 3 was labeled ‘myths and misunderstandings’. This factor only contained items describing myths and misunderstandings.

A three-factor model also provided an adequate description of the data of perceived barriers to palliative care. In the 2013 data, most items with salient loadings on factor 1 contained a variety of policy resources and economics, professional ability and training, messaging and communication, professional knowledge, professional attitudes, patient and family knowledge, and patient and family attitudes. This factor was labeled ‘quality palliative care’. Factor 2 contained items describing how HPs, patients, and families reflected difficulties in implementing palliative care. The factor was labeled ‘palliative care difficulties’. Factor 3 mainly contained items describing issues regarding how patients and families received messages and information from professionals, and communication of HPs with patients and families. Hence, factor 3 was labeled ‘messaging and communication’. In the 2018 data (see Table 5), factor 1 contained items describing information of policy resources and economics, information from HPs, and information about decision making. Factor 1 was labeled ‘messaging and information’. Factor 2 mainly contained items describing knowledge and attitudes of HPs and patients and family members; hence, this factor was labeled ‘knowledge and attitudes’. Factor 3 was labeled ‘quality palliative care’. However, the patterns differed from factor 1 in 2013 since this factor mainly contained items describing labor resources and capital resources.

The total score of knowledge of palliative care in 2013 was significantly lower than the total score in 2018 (*t*= −2.21, *p* = 0.027; see Table 6). As for each factor of knowledge of palliative care scale, the item mean scores of factor 1 and factor 3 in 2013 were significantly lower than the scores in 2018 (*t* = −3.22, *p* = 0.001; *t* = −5.98, *p* < 0.001, respectively). In addition, the total score of perceived barriers to palliative care in 2013 was higher than the total score in 2018 (*t* = 4.46, *p* < 0.001). As for each factor of the perceived barriers scale, the item mean scores of factor 2 in 2013 were higher than those scores in 2018 (*t* = 7.04, *p* < 0.001).

## 4. Discussion

Elevating HPs’ knowledge of palliative care and evaluating how these perceived barriers have changed or persisted over time can help better develop mitigating strategies—including opportunities for education, staffing, and changes in institutions and care systems. Assessing these barriers creates opportunities for further research to address the most critical aspects in improving end-of-life care for patients and their families. Evidence has demonstrated the benefits of palliative care for patients, and understanding knowledge and perceived barriers to palliative care in hospitals is important to expanding access and acceptance of this specialized care for critically ill patients and their families [18]. HPs act as mediators between patients and their families, and between patients and their healthcare teams. A paper found that 79% of participants would use hospice if it were recommended by their physician [19]. Additionally, nurses help families be more aware of patients’ views and concerns, and view the situation more realistically [20,21]. Interestingly, we found that the higher the level of education, the higher the scores of HPs for perceived barriers in 2018. HPs with a graduate degree perceived greater barriers than providers with a university or junior college degree. Because most providers with a graduate degree were head or specialist nurses and they have been consciously responsible for promoting the PAA, they perceived those barriers as higher. It is recommended that PAA- and ACP-related training be included in annual mandatory continuing education for HPs [18,22]. In particular, HPs with managerial roles must undergo educational training of coordination or multidisciplinary consultations [22,23].

Compared to 2013, total scores in 2018 of perceived knowledge of palliative care of HPs had significantly increased (*p* = 0.027). Taking the factors of ‘myths and misunderstandings’ as an example, in the 2013 survey, HPs’ informed consent and advanced decision-making instructions for patients and their families were classified as ‘myths and misunderstandings’. Physicians themselves are still troubled by the need to ‘describe the terminal illness’ to the patient. Physicians think that ‘the doctor needs to save the patient’s life’ instead of allowing the patient to face death. In the early stages of an illness, HPs rarely discuss and communicate ‘about death’ with patients and their families. It is difficult for physicians to predict life expectancy, and they may lack an understanding of patient eligibility guidelines; it is difficult for HPs to explain the patient’s condition and give informed consent [13]. For factor 3, classified as ‘myths and misunderstandings’, the knowledge improvement in 2018 was significantly higher than that in 2013. Facing the wrong concept of palliative care, the concept was clarified in 2018 as compared with 2013. This may indicate that the attitude towards palliative care is gradually improving among HPs. According to the PAA, patients can sign a medical decision form expressing whether they accept or reject receiving medical treatment beforehand when they still maintain full civil capacity [9]. The PAA addresses informed consent and decision making and allows patients to decide whether or not they receive medical treatments at the end stage of a disease. Thus, the ACP advocates the importance of communication in shared decision-making for patients who have a critical illness or who are known to be moving towards the end of life. It is defined as a voluntary discussion between a patient and his/her care provider(s) and family, drawing on a person’s values, goals, and concerns, and any preferences for particular treatments [24]. The process of discussing, consulting, and documenting the wishes and preferences of future care, where possible, offering patients the opportunity to discuss important personal issues, is considered an important improvement in care quality. In this study, the perceived knowledge of HPs also showed that the PAA has had a great effect. In 2018, through the implementation of PAA, the role of informed consent and decision making in palliative care became more prominent, and the knowledge progress of ‘thought and treatment’ represented by factor 1 showed a positive attitude towards ACP.

In 2013, the total score of barriers to palliative care perceived by HPs was significantly higher than that in 2018 (*p* < 0.001); in particular, factor 2, labeled as ‘palliative care difficulties’, in 2013 was significantly higher than factor 2, labeled as ‘knowledge and attitudes’, in 2018. In the 2013 survey, HPs faced many difficulties, including a lack of confidence in promoting palliative care, avoidance of talking about death and related issues, etc. Since the enactment of the decree, we observed that the proportion of certain HPs who have experienced obstacles has changed. Health policy governments announced the participation in hospice as a basic human right [25]. The government cooperates with the instructions and promotion of the PAA, and HPs must participate in PAA training to qualify for certification. Most hospitals have hospice wards, hospice palliative home care, and shared care in order to improve utilization of palliative care. This has had a positive impact, and utilization is rising year to year. However, there are still many difficulties which need solutions. After the promotion of the PAA, these difficulties transited into knowledge and attitudes that HPs must possess, but education and training can be used to help HPs cope with these difficulties. As for dimensions of information and communication between the 2013 and 2018 surveys, end-of-life care, misunderstanding palliative care, information on participation in decision making, and pretestamentary legal factors still plague HPs, patients, and their families. However, the PAA ensures that patients have the right to know all about their condition, and then they can make a decision before the end of life. Promotion of the PAA has encouraged information sharing and communication among HPs, patients, and families.

As for changes over the last 5 years, HPs have reduced implementation barriers through PAA training. Shared decision making (SDM) is seen as a key component of patient-centered care and emphasizes the importance of physician–patient relationships in optimizing health outcomes [26]. To aid the progress of the PAA, SDM can act as an important tool for HPs to discuss the life decision making process with patients and their families. HPs’ ability to face possible obstacles and facilitate decision making requires staff confidence in end-of-life discussions when working with patients and their families while respecting the influence of filial piety, a central value in traditional Chinese culture [27]. One study indicated that participants with high (vs. low) actual knowledge were less likely to find palliative care fear-inducing or depressing and more likely to believe it offered hope [28]. It is essential that palliative care professionals perceive themselves as potential influencers and explicitly transmit the reasons for their intervention [29] while avoiding perpetuating myths, misunderstandings, and positive associations with a lack of palliative care. HPs should grasp the chance to carry out these sharing procedures when a decision is required.

### Strengths and Limitations

This is the first study in Asia to compare knowledge and perceived barriers among HPs before and after promotion of the PAA. Three factors of knowledge and perceived barriers identified can help HPs and stakeholders focus on them and develop interventions. Informed consent and decision making were seen as ‘myths and misunderstandings’ in 2013 but changed to being a part of ‘philosophy and treatment’ in 2018. Accordingly, stakeholders may provide educational training in this direction. There are some limitations to this study. The survey was conducted in two local teaching hospitals in central and northern Taiwan. Even though the two hospitals were of comparable size and had similar properties, there might still be differences between them. The results should be interpreted with caution. Additionally, although respondents of physicians and nurses represented a wide range of experiences in local hospitals, the surveys did not specifically investigate physicians and nurses working in palliative care. Compared to the general HPs, this group may have different levels of knowledge of palliative care and encounter distinct barriers to palliative care before and after promotion of the PAA. Moreover, the study did not include other HPs, such as social workers or psychotherapists. The results might not can be generalizable to physicians and nurses working in palliative care or other HPs. Future research should be conducted on physicians and nurses working in palliative care and other HPs.

## 5. Conclusions

Our study investigated changes in knowledge and perceptions of barriers of palliative care over 5 years before and after promoting the PAA. This study showed that after the PAA policy was promoted, HPs’ knowledge of palliative care increased while the perceived barriers decreased. Although there are still some obstacles, such as soft and hard resources, the government should provide more on-the-job education for HPs on palliative care. In addition, HPs need to be aware of improvements in policies and regulations and assist patients in a timely manner. Moreover, future studies should examine whether there are differences in knowledge and barrier perceptions of palliative care in different types of HPs. Additional studies should be conducted to further understand the barriers HPs face and what strategies should be developed to ensure that the challenges impeding palliative care as well as the PAA’s implementation are addressed.

## Figures and Tables

**Table 1:** Characteristics of participants in 2013 and 2018.

	Year 2013 *N* = 277		Year 2018 *N* = 222	
Variable	Mean (SD)	*n* (%)	Mean (SD)	*n* (%)
Age (years)	33.2 (8.1)		32.1 (8.5)	
Knowledge of palliative care	112.0 (15.0)		115.0 (14.6)	
Perceived barriers to palliative care	106.4 (18.2)		99.6 (15.8)	
Gender				
Female		220 (79.4)		175 (78.8)
Job tenure (years)				
<1		1 (0.4)		17 (7.7)
1~2		45 (16.2)		42 (18.9)
3~4		48 (17.3)		31 (14.0)
5~6		29 (10.5)		28 (12.6)
7~10		54 (19.5)		30 (13.5)
>11		100 (36.1)		74 (33.3)
Marital status				
Married		135 (48.7)		109 (49.1)
Unmarried		140 (50.2)		111 (50.0)
Divorced		2 (0.7)		2 (0.9)
Educational level				
Junior college		85 (30.6)		74 (33.3)
Bachelor’s degree		174 (62.8)		137 (61.7)
Graduate school		18 (6.5)		11 (5.0)
Working unit				
Medical/surgical ward		162 (58.5)		110 (49.5)
Emergency/intensive care unit		71 (25.6)		85 (38.3)
Other		44 (15.9)		27 (12.2)

Abbreviation: SD, standard deviation.

**Table 2:** Predictive variables of knowledge of palliative care in 2013 and 2018.

	Year 2013				Year 2018			
Variable	B	SE	*t*	*p*	B	SE	*t*	*p*
Age	−0.001	0.006	−0.17	0.87	−0.007	0.006	−1.13	0.26
Job tenure	0.006	0.028	0.20	0.84	0.002	0.029	0.08	0.93
Educational level (graduate school as the reference)								
Junior college	0.024	0.128	0.19	0.85	−0.309	0.142	−2.18	0.03
Bachelor’s	0.015	0.115	0.13	0.90	−0.298	0.134	−2.23	0.03
Marital status (divorced as the reference)								
Unmarried	0.353	0.308	1.14	0.25	−0.506	0.302	−1.68	0.09
Married	0.415	0.313	1.33	0.19	−0.489	0.299	−1.64	0.10
Gender (male as the reference)								
Female	−0.145	0.086	−1.68	0.09	0.155	0.080	1.93	0.05
Working unit (other as the reference)								
Medical/surgical ward	0.057	0.074	0.76	0.45	0.038	0.091	0.42	0.68
Emergency/ICU	0.158	0.83	1.89	0.06	−0.0004	0.093	0	1.00

Abbreviation: ICU, intensive care unit; B, unstandardized coefficient; SE, standard error.

**Table 3:** Predictive variables of perceived barriers to palliative care in 2013 and 2018.

	Year 2013				Year 2018			
Variable	B	SE	*t*	*p*	B	SE	*t*	*p*
Age	−0.019	0.009	−2.21	0.03	0.001	0.008	0.14	0.89
Job tenure	0.119	0.036	3.29	0.001	0.038	0.034	1.12	0.26
Educational level (graduate school as the reference)								
Junior college	−0.155	0.164	−0.94	0.35	−0.562	0.166	−3.39	0.0008
Bachelor’s	−0.076	0.147	−0.52	0.61	−0.376	0.157	−2.4	0.02
Marital status (divorced as the reference)								
Unmarried	0.398	0.395	1.01	0.32	−0.308	0.353	−0.87	0.38
Married	0.505	0.401	1.26	0.21	−0.413	0.349	−1.18	0.23
Gender (male as the reference)								
Female	0.051	0.157	0.32	0.74	0.108	0.164	0.66	0.51
Working unit (others as the reference)								
Medical/ surgical ward	0.031	0.095	0.33	0.74	−0.016	0.107	−0.15	0.88
Emergency/ICU	0.121	0.107	1.13	0.26	0.025	0.109	0.23	0.82

Abbreviation: ICU, intensive care unit; B, unstandardized coefficient; SE, standard error.

**Table 4:** Factor loadings for items of the scale of knowledge of palliative care in 2013 and 2018.

Variable	Year 2013			Year 2018		
	Factor 1	Factor 2	Factor 3	Factor 1	Factor 2	Factor 3
24. Healthcare providers’ attitudes towards palliative care influence patients’ willingness to receive palliative care.	0.84			0.80		
21. Difficulties for healthcare providers when caring for end-of-life patients are pain management, lack of mental support ability, and provider stress.	0.82			0.57		
22. End-of-life patients feel confused, helpless, difficult, and tired at the beginning of the decision-making process of receiving palliative care.	0.80			0.64		
28. The needs of end-of-life patients in palliative care are physical symptom management, family care, diagnostic disclosure, and a peaceful death.	0.79			0.46		
25. The more information that healthcare providers provide, the more helpful they are in assisting patients and families in decision making.	0.76			0.71		
26. It is not necessary for patients receiving palliative care to stay in a hospice ward; they can receive hospice shared care or hospice home care.	0.68			0.61		
6. Palliative care is not only for cancer patients, but also for other end-of-life patients based on new regulations.	0.67				0.49	
23. The reason for end-of-life patients to receive palliative care is that they want to have a peaceful death.	0.63			0.61		
9. Hospice wards in hospitals are for the acute care of end-of-life patients; hence, stable end-of-life patients can choose hospice home care.	0.62			0.32		
20. The healthcare provider is the main person that refers patients to palliative care. Patients often have a delayed referral; if the referral occurs earlier, the quality of life of the patient can be improved.	0.57			0.64		
27. Most patients and families have misunderstandings about palliative care, such as the idea of the hospice ward as a place to wait for death.	0.53				0.41	
5. Most patients and families lack information regarding how palliative care can improve the quality of life of end-of-life patients.	0.52				0.53	
30. Healthcare providers can be made aware of their feelings about death through caring for end-of-life patients.	0.42			0.60		
18. Whether healthcare providers have experience in taking care of end-of-life patients can influence a patient’s and their family’s willingness to receive palliative care.	0.31			0.63		
Palliative care provides humanistic and holistic nursing care to incurable patients.	0.30				0.41	
4. For dying patients, palliative care can alleviate pain and symptoms and provide comfort care.		0.77			0.58	
3. End-of-life patients receive palliative care because their identification of death is a natural process that does not accelerate or prolong death.		0.75			0.54	
12. Signing a ‘do not resuscitate’ form should be based on the patient’s opinion, not the family’s opinion.		0.58			0.50	
16. After the end-of-life patient receives palliative care, in addition to helping the patient with symptom relief, it is more important to assist the patient and his/her family to understand the conditions and fulfill the four principles of life (love, gratitude, forgiveness, and grace in bidding farewell).		0.50			0.43	
19. Family members are the most important support force for terminally ill patients. Therefore, receiving palliative care can obtain the care of the whole person, the whole family, the whole process, and the whole team to help the patient through the final process and grief of the patient's life.		0.47		0.47		
17. Existing regulations strictly regulate removal of a terminal patient’s life-saving machine. If the patient or family member submits an application, it must be reviewed by two specialist physicians and a hospital ethics review meeting.		0.46		0.47		
14. Knowing the patient’s condition and medical treatment at the end of the period can help the patient choose to receive palliative care.		0.44			0.53	
11. If the patient’s rights are not taken seriously, the nurse is responsible for discussing the medical treatment with the physician and other medical team members.		0.41			0.54	
15. Pain control for end-of-life patients is based on the principle of reducing pain; the patient need not fear painkiller addiction.		0.41			0.59	
29. The purpose of the patient’s hospitalization for palliative care is because the family cannot take care of the patient and wants to save medical expenses by withholding treatment.			0.58			0.56
10. The best time for a patient to stay in the hospice ward is when the patient is dying.			0.54			0.55
2. For dying patients (including cancer and noncancer causes), the ‘do not resuscitate’ mechanism is an inhumane act of death.			0.49			0.49
13. End-of-life patients receiving palliative care have to pay a higher cost than those receiving care in the general ward.			0.49			0.49
8. One of the factors affecting a patient’s admission to the palliative care ward is that the patient or family believes that death is the only way to discharge.			0.38		0.62	
7. The patient and family are afraid that the doctor’s choice of palliative care means giving up on the patient.			0.37		0.62	

**Table 5:** Factor loadings for items of the scale of perceived barriers to palliative care in 2013 and 2018.

	Year 2013			Year 2018		
Variable	Factor 1	Factor 2	Factor 3	Factor 1	Factor 2	Factor 3
22. The hospital has insufficient manpower and equipment.	0.81					0.60
31. The number of beds in the palliative care ward is insufficient.	0.77					0.68
26. For healthcare providers, spending too much time on taking care of end-of-life patients is a big obstacle.	0.75					0.38
23. The hospital has not established a palliative care ward.	0.75					0.63
21. The hospital does not pay attention to palliative care.	0.72				0.42	
24. Health insurance payments for the palliative care ward are not cost-effective.	0.70				0.36	
30. Physicians cannot provide efficient referral services.	0.70					0.50
20. Education and training of healthcare providers are insufficient.	0.65				0.55	
27. Dealing with patient emotions is a big challenge.	0.64					0.51
18. Healthcare providers have insufficient capacity for terminal care.	0.63				0.75	
32. The patient does not understand his/her condition.	0.63					0.48
16. Healthcare providers lack awareness of palliative care.	0.62				0.79	
28. Family decision making has no consensus.	0.61			0.58		
13. Hospice home care is provided without 24 h access.	0.58			0.45		
17. Healthcare providers have misunderstandings about palliative care.	0.57				0.81	
29. The insurance company does not pay the fee for staying in the palliative care ward.	0.57				0.38	
25. Primary healthcare providers are unable to make decisions since they are not major decision makers.	0.56			0.35		
12. Healthcare providers are too busy to have enough time to listen to patient needs because they are taking care of too many patients.	0.54			0.57		
19. Healthcare providers lack confidence in promoting palliative care.	0.30				0.63	
2. Patients, their families, and healthcare providers still often avoid talking about death or related issues.		0.76			0.39	
Discussing issues related to the end of life is hard to do for healthcare providers.		0.75			0.51	
3. Healthcare providers are afraid that they are unable to deal with the emotional reactions of patients after they inform them about their condition.		0.73			0.37	
4. Healthcare providers begin discussions when a patient’s condition deteriorates.		0.58		0.39		
15. Palliative care is not in line with my own philosophy. I want to advise patients and their families to engage in active treatment.		0.45			0.67	
14. Healthcare providers’ attitudes towards palliative care are negative.		0.37			0.71	
7. Patients do not feel that it is important to take the initiative to participate in decision making.		0.36		0.65		
5. Patients and their families are insufficiently aware about palliative care.			0.60	0.77		
6. Patients and their families have misunderstandings about palliative care.			0.57	0.77		
8. Patients and their families lack information on end-of-life care.			0.54	0.84		
9. Healthcare providers do not clearly understand the law of pretestamentary wills.			0.39	0.75		
11. The medical team does not have good communication with patients and their families.			0.38	0.56		
10. The patient has been unable to express an opinion on treatment.			0.33	0.57		

**Table 6:** Evaluations of mean scores of the total scale and the factors of knowledge of palliative care scale and perceived barriers to palliative care in 2013 and 2018.

	Year 2013 Mean (SD)	Year 2018 Mean (SD)	*t* (*p*)
Knowledge of palliative care			
Total score	112.0 (15.0)	115.0 (14.6)	−2.21 (0.027)
Item mean score for factor 1, policies, regulations, and promotion	3.70 (0.58)	3.86 (0.58)	−3.22 (0.001)
Item mean score for factor 2, philosophy and treatments	3.84 (0.64)	3.76 (0.59)	1.48 (0.141)
Item mean score for factor 3, myths and misunderstandings	3.66 (0.47)	3.96 (0.61)	−5.98 (<0.001)
Perceived barriers to palliative care			
Total score	106.4 (18.2)	99.6 (15.8)	4.46 (<0.001)
Item mean score for factor 1, quality palliative care (Year 2013) factor 1, messaging and information (Year 2018)	3.35 (0.63)	3.29 (0.57)	1.22 (0.222)
Item mean score for factor 2, palliative care difficulties (Year 2013) factor 2, knowledge and attitudes (Year 2018)	3.18 (0.62)	2.81 (0.55)	7.04 (<0.001)
Item mean score for factor 3, messaging and communication (Year 2013) factor 3, quality palliative care (Year 2018)	3.41 (0.59)	3.39 (0.65)	0.35 (0.731)

Abbreviation: SD, standard deviation.

## Data Availability

The datasets used and/or analyzed as part of the present study are available from the corresponding author on reasonable request.

## References

[1] Taiwan National Development Council Population Projections of Republic of China (2018–2065). https://www.ndc.gov.tw/Content_List.aspx?n=84223C65B6F94D72.

[2] Tang S.T., Chen M.-L., Huang E.-W., Koong S.-L., Lin G.L., Hsiao S.-C. (2007). Hospice Utilization in Taiwan by Cancer Patients Who Died between 2000 and 2004. J. Pain Symptom Manag..

[3] Mezey M., Mitty E., Rappaport M., Ramsey G. (1997). Implementation of the Patient Self-Determination Act (PSDA) in Nursing Homes in New York City. J. Am. Geriatr. Soc..

[4] McDonald A. (2010). The Impact of the 2005 Mental Capacity Act on Social Workers’ Decision Making and Approaches to the Assessment of Risk. Br. J. Soc. Work..

[5] Lynch T., Connor S., Clark D. (2013). Mapping Levels of Palliative Care Development: A Global Update. J. Pain Symptom Manag..

[6] Yang C.-L., Chiu T.-Y., Hsiung Y.-F.Y., Hu W.-Y. (2011). Which Factors Have the Greatest Influence on Bereaved Families’ Willingness to Execute Advance Directives in Taiwan?. Cancer Nurs..

[7] Chang H.-T., Lin M.-H., Chen C.-K., Chou P., Chen T.-J., Hwang S.-J. (2016). Trends of Do-Not-Resuscitate Consent and Hospice Care Utilization among Noncancer Decedents in a Tertiary Hospital in Taiwan between 2010 and 2014: A Hospital-Based Observational Study. Medicine.

[8] Shih T.-C., Chang H.-T., Lin M.-H., Chen C.-K., Chen T.-J., Hwang S.-J. (2017). Trends of Do-Not-Resuscitate Orders, Hospice Care Utilization, and Late Referral to Hospice Care among Cancer Decedents in a Tertiary Hospital in Taiwan between 2008 and 2014: A Hospital-Based Observational Study. J. Palliat. Med..

[9] The Patient Autonomy Act (PAA) Law & Regulations Database of the Republic of China. https://law.moj.gov.tw/LawClass/LawAll.aspx?pcode=L0020189.

[10] Ministry of Health and Welfare Taiwan’s Dying Patient’s Death Quality Ranks First in Asia, and Continues to Strengthen ‘Palliative Care’ as One of the ‘Integrated Health Care’. https://www.mohw.gov.tw/fp-2627-19127-1.html.

[11] Dong F., Zheng R., Chen X., Wang Y., Zhou H., Sun R. (2016). Caring for Dying Cancer Patients in the Chinese Cultural Context: A Qualitative Study from the Perspectives of Physicians and Nurses. Eur. J. Oncol. Nurs. Off. J. Eur. Oncol. Nurs. Soc..

[12] Lin C.-P., Cheng S.-Y., Chen P.-J. (2018). Advance Care Planning for Older People with Cancer and Its Implications in Asia: Highlighting the Mental Capacity and Relational Autonomy. Geriatrics.

[13] Brickner L., Scannell K., Marquet S., Ackerson L. (2004). Barriers to Hospice Care and Referrals: Survey of Physicians’ Knowledge, Attitudes, and Perceptions in a Health Maintenance Organization. J. Palliat. Med..

[14] Davies B., Sehring S.A., Partridge J.C., Cooper B.A., Hughes A., Philp J.C., Amidi-Nouri A., Kramer R.F. (2008). Barriers to Palliative Care for Children: Perceptions of Pediatric Health Care Providers. Pediatrics.

[15] Kavalieratos D., Mitchell E.M., Carey T.S., Dev S., Biddle A.K., Reeve B.B., Abernethy A.P., Weinberger M. (2014). “Not the ‘Grim Reaper Service’”: An Assessment of Provider Knowledge, Attitudes, and Perceptions Regarding Palliative Care Referral Barriers in Heart Failure. J. Am. Heart Assoc..

[16] Ministry of Health and Welfare Annual Health Education Spindle Project Key Work and Strategy. https://www.mohw.gov.tw/lp-173-1.html.

[17] Chiang C.I., Chen N.W., Huang T.W. (2013). Exploring Nursing Staff Awareness of and Analyzing Barriers to Hospice Care for Terminal Cancer Patients. Cheng Ching Med. J..

[18] Hawley P. (2017). Barriers to Access to Palliative Care. Palliat. Care.

[19] Spruill A.D., Mayer D.K., Hamilton J.B. (2013). Barriers in Hospice Use Among African Americans with Cancer. J. Hosp. Palliat. Nurs..

[20] Black K. (2006). Advance Directive Communication: Nurses’ and Social Workers’ Perceptions of Roles. Am. J. Hosp. Palliat. Care.

[21] Seymour J., Almack K., Kennedy S. (2010). Implementing Advance Care Planning: A Qualitative Study of Community Nurses’ Views and Experiences. BMC Palliat. Care.

[22] Ke L.-S., Huang X., O’Connor M., Lee S. (2015). Nurses’ Views Regarding Implementing Advance Care Planning for Older People: A Systematic Review and Synthesis of Qualitative Studies. J. Clin. Nurs..

[23] Tran M., Grant M., Clayton J., Rhee J. (2018). Advance Care Decision Making and Planning. Aust. J. Gen. Pract..

[24] National Health Service (NHS) (2008). Advance Care Planning: A Guide for Health and Social Care Staff.

[25] Ministry of Health and Welfare Enjoy Hospice as a Basic Human Right. https://www.hpa.gov.tw/Pages/Detail.aspx?nodeid=1128&pid=1950.

[26] Barry M.J., Edgman-Levitan S. (2012). Shared Decision Making--Pinnacle of Patient-Centered Care. N. Engl. J. Med..

[27] Lin C.-P., Evans C.J., Koffman J., Sheu S.-J., Hsu S.-H., Harding R. (2019). What Influences Patients’ Decisions Regarding Palliative Care in Advance Care Planning Discussions? Perspectives from a Qualitative Study Conducted with Advanced Cancer Patients, Families and Healthcare Professionals. Palliat. Med..

[28] Zimmermann C., Wong J.L., Swami N., Pope A., Cheng Y., Mathews J., Howell D., Sullivan R., Rodin G., Hannon B. (2021). Public knowledge and attitudes concerning palliative care. BMJ Support. Palliat. Care.

[29] Reigada C., Centeno C., Gonçalves E., Arantzamendi M. (2021). Palliative Care Professionals’ Message to Others: An Ethnographic Approach. Int. J. Environ. Res. Public Health.

